# Apigenin Induces the Apoptosis and Regulates MAPK Signaling Pathways in Mouse Macrophage ANA-1 Cells

**DOI:** 10.1371/journal.pone.0092007

**Published:** 2014-03-19

**Authors:** Yuexia Liao, Weigan Shen, Guimei Kong, Houning Lv, Wenhua Tao, Ping Bo

**Affiliations:** 1 Department of Chinese Medicine Integrated with Western Medicine, Yangzhou University School of Medicine, Yangzhou, P.R. China; 2 Department of Cell Biology, Yangzhou University School of Medicine, Yangzhou, P.R. China; 3 Department of Medicine, Yangzhou University School of Medicine, Yangzhou, P.R. China; UAE University, Faculty of Medicine & Health Sciences, United Arab Emirates

## Abstract

Apigenin is a naturally occurring plant flavonoid that possesses antioxidant, anti-cancer and anti-inflammatory properties. However, there are few reports has been done on the ability of apigenin to induce apoptosis in macrophages. In this study, mouse macrophage ANA-1 cells were incubated with different concentrations of apigenin. The cell viability was determined by an MTT assay. The cell apoptosis were analyzed by flow cytometric analysis. Apoptosis were also analyzed using a TUNEL assay and a DNA ladder. The level of intracellular ROS was detected using a dichlorofluorescein -diacetate probe. The expression levels of apoptosis-related proteins were detected by western blot analysis. The results showed that apigenin decreased the viability of ANA-1 cells and induced apoptosis in a dose- and time-dependent manner. Apigenin increased the level of intracellular ROS, downregulated the expression of Bcl-2 and upregulated the expression of caspase-3 and caspase-8 in ANA-1 cells. Furthermore, apigenin downregulated the expression of phospho-ERK and phospho-JNK, upregulated the expression of phospho-p38 and had no significant effect on the expression of Bax, ERK, JNK and p38. The results suggested that apigenin induced cell apoptosis in mouse macrophage ANA-1 cells may via increasing intracellular ROS, regulating the MAPK pathway, and then inhibiting Bcl-2 expression.

## Introduction

Apigenin, also known as 4′,5,7,-trihydroxyflavone, is a natural plant flavonoid that is abundantly present in common fruits, vegetables, beans, teas, herbs and wines or beer that are brewed from natural ingredients and is recognized as a bioactive flavonoid that has been shown to possess antioxidant, anti-cancer and anti-inflammatory properties [1], [2]. Previous studies have shown that apigenin possesses antioxidant and scavenging free radicals effects in vitro as well as in vivo and can alleviate kainic acid-induced excitotoxicity by quenching ROS in hippocampal neurons [3], [4]. Epidemiologic studies have revealed that a diet rich in apigenin decreases the risk of certain cancers [4]. The evidence from other studies has shown that apigenin can inhibit cancer cell growth via the promotion of cell cycle arrest or apoptosis [5], [6]. Meanwhile, several studies have also shown that apigenin has a potential regulatory effect on inflammatory reactions that are mediated by mast cells and inhibits the expression of inflammatory factors (such as IL-6, IL-8 and ICAM-1) in human umbilical vein endothelial cells [7], [8]. These findings suggest that apigenin has anti-inflammatory and anti-cancer activity and may be a therapeutic strategy for cancer and inflammatory diseases.

Macrophages are important residents in all tissues and are central mediators of the immune system that contribute to the initiation and resolution of inflammation and that regulate tissue homeostasis; additionally, macrophages are critically involved in diseases that are caused by chronic inflammation (e.g., arthritis, multiple sclerosis, diabetic ulcers, inflammatory bowel diseases, cardiovascular disease) [9], [10], [11], [12], [13]. In solid tumors, macrophages are also major determinants of immune suppression [9]. High macrophage density has been primarily associated with the poor prognosis of cancer patients and with resistance to therapies [14]. Meanwhile, in tumor ecosystems, macrophages are the most abundant innate immune cells and are the key initiators of subtle chronic inflammation in the tumor microenvironment [15]. Tumor-associated macrophages, which are the key promoters of cancer-related inflammation, promote the initiation and malignant progression of cancer and represent a predominant population of inflammatory cells that are present in solid tumors and that play an important role in tumor growth, angiogenesis, metastasis, matrix remodeling and immune evasion in human and animal tumors [14], [16]. Macrophages are also the initiators and regulators of different inflammatory diseases; macrophages can be recruited by the release of cytokines and then guide the course of inflammation [10]. Promoting macrophage apoptosis and/or eliminating activated macrophages has been proven to be a promising way of resolving inflammation in animal models and a beneficial therapeutic strategy for inflammatory diseases, such as asthma, rheumatoid arthritis and inflammatory bowel disease [10], [17]. Taken together, these findings suggested that suppressing the survival of macrophages or inducing the apoptosis of macrophages might be a key component to preventing and possibly treating macrophage-related inflammatory diseases and cancer [14], [18].

Although apigenin is effective at preventing the onset of inflammation and cancer, it is unclear whether apigenin exerts anti-inflammatory and anti-cancer effects through a macrophage-related therapeutic strategy. There are few reports has been done on the ability of apigenin to induce apoptosis in macrophages. In the present study, the results shown that apigenin inhibited the cell viability of mouse macrophage ANA-1 cells via inducting apoptosis. The paper aimed to explore the mechanism of apignein induced ANA-1 cell apoptosis and related proteins expression.

## Materials and Methods

### Reagents and antibodies

Apigenin (HPLC>98%) was purchased from the Nanjing TCM Institute of Chinese Materia Medica and was dissolved in sodium carbonate (20 mM). Purified mouse anti-Bax and anti-Bcl-2 antibodies were purchased from Biolegend (USA). Mouse monoclonal anti-phospho-ERK1/2, anti-caspase-3, anti-caspase-8, anti-phospho-p38, rabbit monoclonal anti-ERK1/2, anti-p38, mouse monoclonal anti-β-actin, rabbit/goat anti-mouse IgG and goat anti-rabbit IgG antibodies were purchased from Santa Cruz Biotechnology (USA). Mouse monoclonal anti-phospho-JNK and anti-JNK antibodies were purchased from Cell Signaling Technology (USA). Apoptosis, DNA Ladder Extraction Kit with Spin Column, DNA Molecular Weight Marker was purchased from Beyotime Institute of Biotechnology (Shanghai, China). In Situ Cell Death Kit, Fluorescein was purchased from Roche Applied Science, Mannheim, Germany.

### Cell Culture

ANA-1 macrophages were obtained from the Cell Bank of the Shanghai Institute of Biochemistry and Cell Biology, Chinese Academy of Sciences (Shanghai, China) and were cultured in RPMI 1640 medium, which was supplemented with 10% fetal bovine serum (FBS), 100 units/ml penicillin and 100 μg/ml streptomycin at 37°C in a fully humidified incubator containing 5% CO_2_.

### Cell Viability Assay

The MTT (3-(4,5-dimethylthiazol-2-yl)-2,5-diphenylterazolium bromide) assay was used to detect the viability of cells. Briefly, ANA-1 cells were cultured in 96-well culture plates (2×10^5^ per well) that contained 200 μl of medium with 0, 12.5, 25, 50, 100, or 200 μM apigenin for 24 h or 48 h and were then incubated with 20 μl of PBS containing 5 mg/ml MTT for 4 h at 37°C in 5% CO_2_. Next, the MTT solution was removed from the wells by aspiration, and the formazan crystals were dissolved in 100 μl of DMSO. The absorbance was measured at 570 nm, with a reference at 470 nm, using a Bio-Tek MQX 680 (Bio-Tek Instruments Inc., Winooski, VT, USA). The data are expressed as the mean ± the standard deviation of the mean (SD) for at least five independent experiments.

### Apoptosis Assay

The induction of apoptosis in ANA-1 cells by apigenin was determined by (a) the quantification of cells in sub-G_1_ DNA content by flow cytometry following staining with propidium iodide (PI); (b) the quantification of apoptotic cells by an Annexin V-FITC Apoptosis Detection Kit (KeyGen Biotech Co. Roche, Nanjing, China); and (c) western blot showing the levels of caspase-3, caspase-8, Bax and Bcl-2. For the analysis of sub-G_1_ DNA content, the ANA-1 cells were directly treated with the indicated concentrations of apigenin for 24 h, fixed in ethanol (70%) at 4°C and stained with PI (50 μg/mL). The number of apoptotic cells was quantified by measuring the sub-G_1_ population by flow cytometry (BD Biosciences, San Jose, CA, USA) using Cell Quest Software. Apoptosis was also quantified by Annexin V-FITC and PI dual staining using an Annexin V-FITC Apoptosis Detection Kit according to the manufacturer's instructions. The levels of caspase-3, caspase-8, Bax and Bcl-2 were determined by western blot analysis as described below.

### TUNEL assay

ANA-1 Cells were treated with apigenin or vehicle for 48 h and then fixed with 4% paraformaldehyde and permeabilized with 0.1% sodium citrate and 0.1% Triton X-100. DNA fragmentation was determined by *In Situ Cell Death Kit, Fluorescein* (Roche Applied Science, Mannheim, Germany) following the manufacturer's instruction. TUNEL positive apoptotic ANA-1 cells captured using confocal microscopy and flow cytometry.

### DNA fragmentation assay

The DNA fragmentation analysis was performed as described using DNA ladder Extraction kit. The ANA-1 cells were cultured in the presence or absence of apigenin for 48 h, then gently harvested by centrifugation. The cells were incubated for 20 min in lysis buffer on ice. After centrifugation, the soluble DNA fragments were dissolved in TE and loaded onto a 1.5% agarose gel and separated at 50 V for 150 min. The DNA fragments were visualized by transillumination under UV light.

### ROS measurement

The level of intracellular ROS was detected using fluorescence microscope and a dichlorofluorescein- diacetate (DCF-DA) probe (Beyotime Institute of Biotechnology, Shanghai, China). The ANA-1 cells (5×10^5^/ml) were plated on 6-well culture plates and treated with apigenin (0, 12.5, 25, 50, 100, 200 μM) for 24 h. After treatment, cells were rinsed with PBS solution and incubated in a medium containing 10 μM DCF-DA for 45 min at 37°C in a dark environment. The DCF-DA loaded cells were then rinsed with PBS solution and observed by using fluorescence microscopy. Fluorescence was measured at an excitation wavelength of 480 nm and an emission wavelength of 520 nm.

### Western blot analysis

The cells were lysed in cell lysis buffer (Beyotime Institute of Biotechnology, Shanghai, China) containing 1 μmol/L phenylmethyl-sulfonyl fluoride, 1.5 μM pepstatin A and 0.2 μM leupeptin. Protein concentrations were quantified by the Bradford assay. The samples were separated by 10% SDS-polyacrylamide gel electrophoresis and were then transferred to a polyvinylidene fluoride membrane, which was blocked with 5% dried milk protein; after blocking, the blots were incubated with monoclonal antibodies. After being washed with TBST, the membranes were incubated at 37°C for 1 h in blocking buffer that contained the secondary antibodies, which included goat anti-rabbit IgG or rabbit anti-mouse IgG, and were visualized by a chemiluminescence ECL western blotting analysis system (BD, USA). The protein levels were quantified using ImageJ software (NIH) and were expressed as percentages of the control after being normalized with the housekeeping protein β-actin.

### Statistical analysis

All of the data represent at least 3 independent experiments and were expressed as the mean ± SD unless otherwise indicated. Statistically significant differences between the groups were assessed through independent simple *t-*tests or one-way ANOVA when multiple comparisons against the control were required. *P*-values that were less than 0.05 were considered significant.

## Results

### 1. Apigenin decreases ANA-1 cell viability

**Figure 1:**
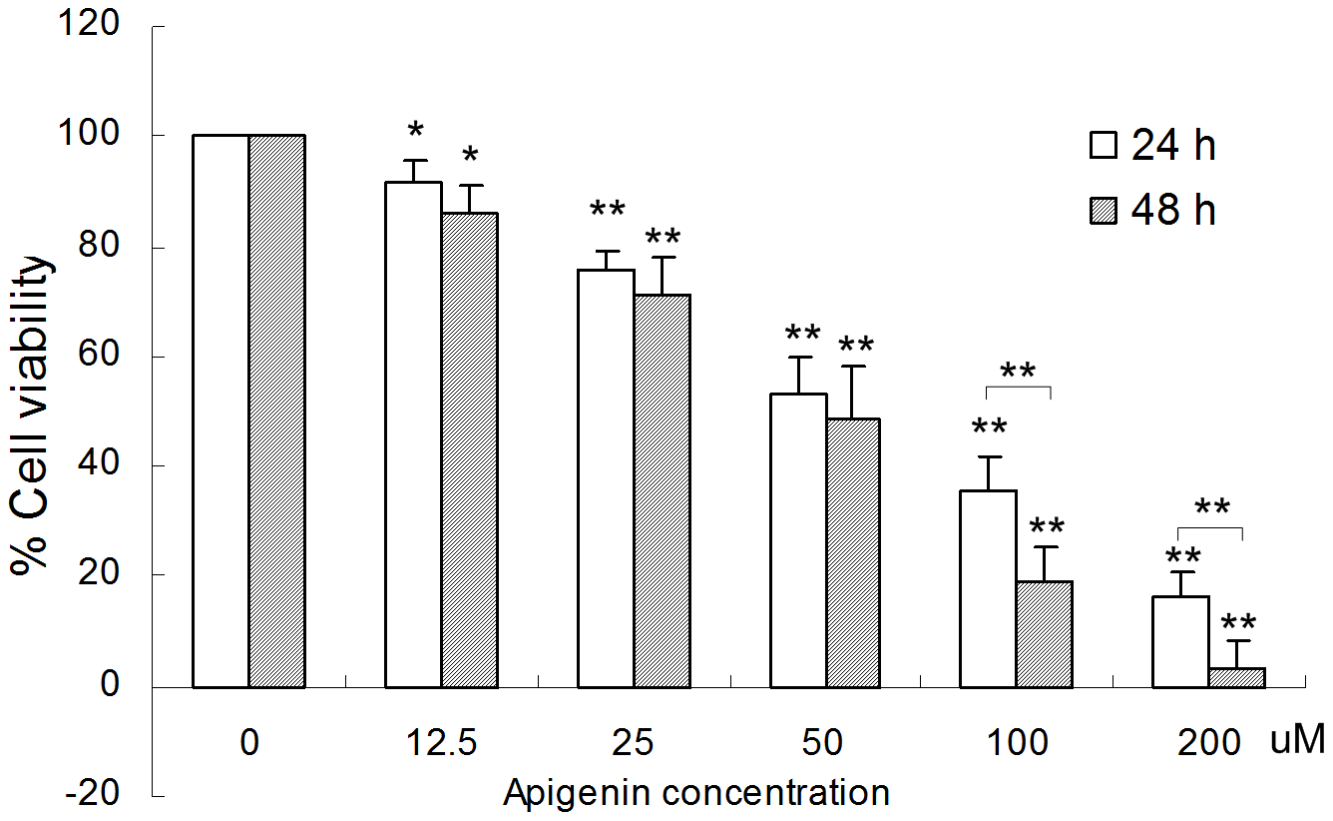
Apigenin inhibits the viability of ANA-1 cells. The ANA-1 cells were exposed to either vehicle (2 mM Na_2_CO_3_ in medium) or apigenin and were incubated as described above. Cell viability was analyzed by the MTT assay. All data are expressed as the percentage of change in comparison with the control group, which were assigned 100% viability. The values are given as the mean ± SD. *p<0.05, **, *p*<0.01, versus the control.

To analyze the effect of apigenin on the viability of ANA-1 cells, the cells were exposed to different concentrations (0, 12.5, 25, 50, 100, 200 μM) of apigenin for 24 h or 48 h. The results from the MTT assay showed that apigenin significantly decreased the viability of ANA-1 cells in a dose- and time-dependent manner (Fig. 1). After a 48 h incubation with 50 μM apigenin, the viability of the cells was reduced by 47%. Moreover, 200 μM apigenin reduced cell viability by 89% or more after a 24 h or 48 h treatment. The IC_50_ for apigenin was approximately 50 μM for the ANA-1 cells. These results indicate that apigenin decreases the viability of ANA-1 cells.

### 2. Apigenin induces ANA-1 cell apoptosis

**Figure 2:**
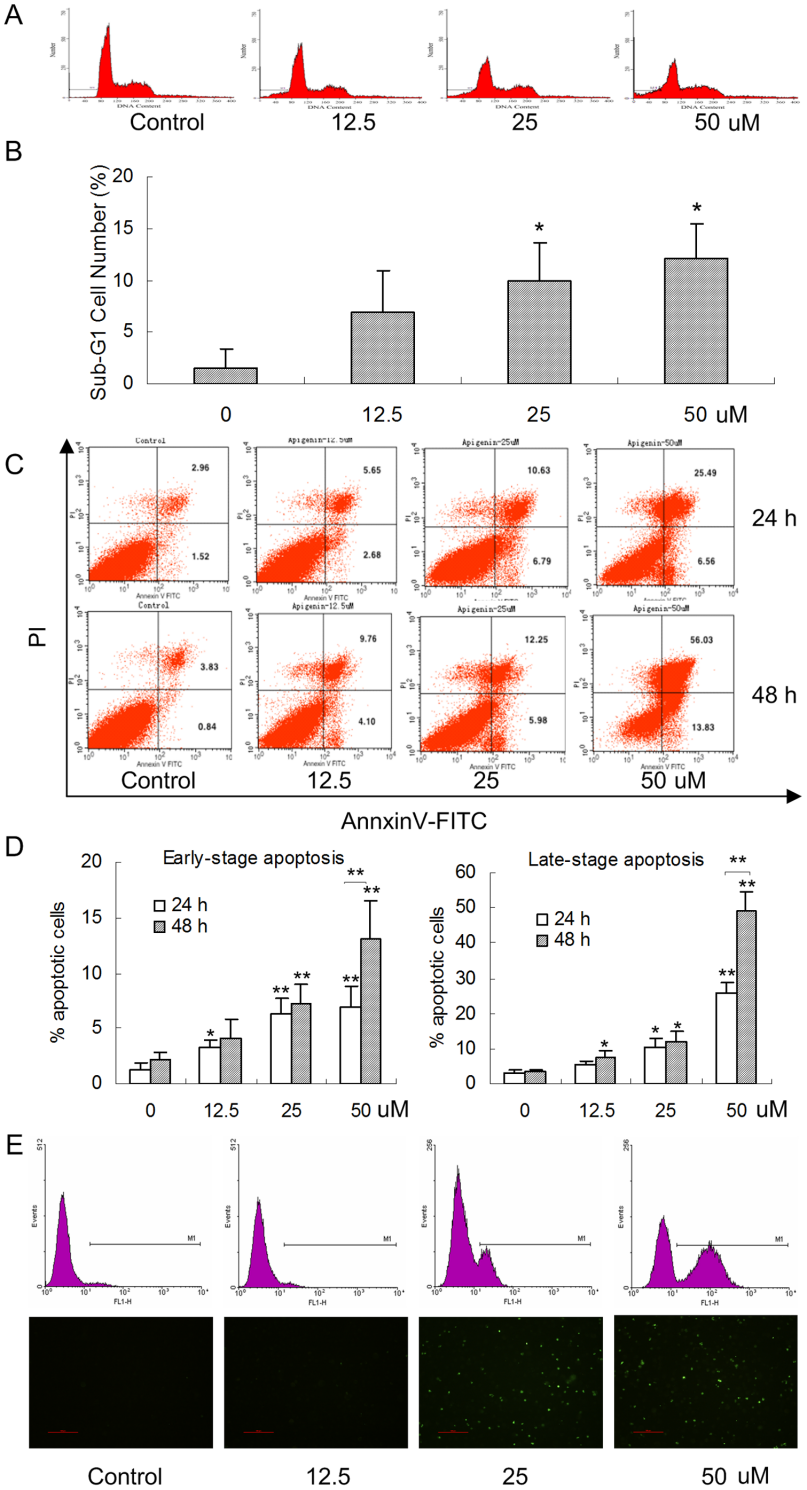
Apigenin induces the apoptosis of ANA-1 cells. A. The quantification of flow cytometry analyses. The flow cytometry data showed one representative sub-G_1_ result. B. The apoptotic rates are given as the percentage of cells with sub-G_1_ DNA content. The values are given as the mean ± SD. *, *p*<0.05, versus the control. C. ANA-1 macrophages were exposed to different concentrations of apigenin for 24 h and 48 h. The flow cytometry data showed one representative dual staining result. D. Early- (%) and late-stage (%) apoptotic cell death were detected by staining cells with Annexin V-FITC and PI and analyzing by flow cytometry. The values are given as the mean ± SD. *, *p*<0.05, **, *p*<0.01, versus the control. E. the ANA-1 cells were exposed to apigenin for 48 h. The images of TUNEL positive cells were captured by flow cytometry and a confocal microscopy (×200).

**Figure 3:**
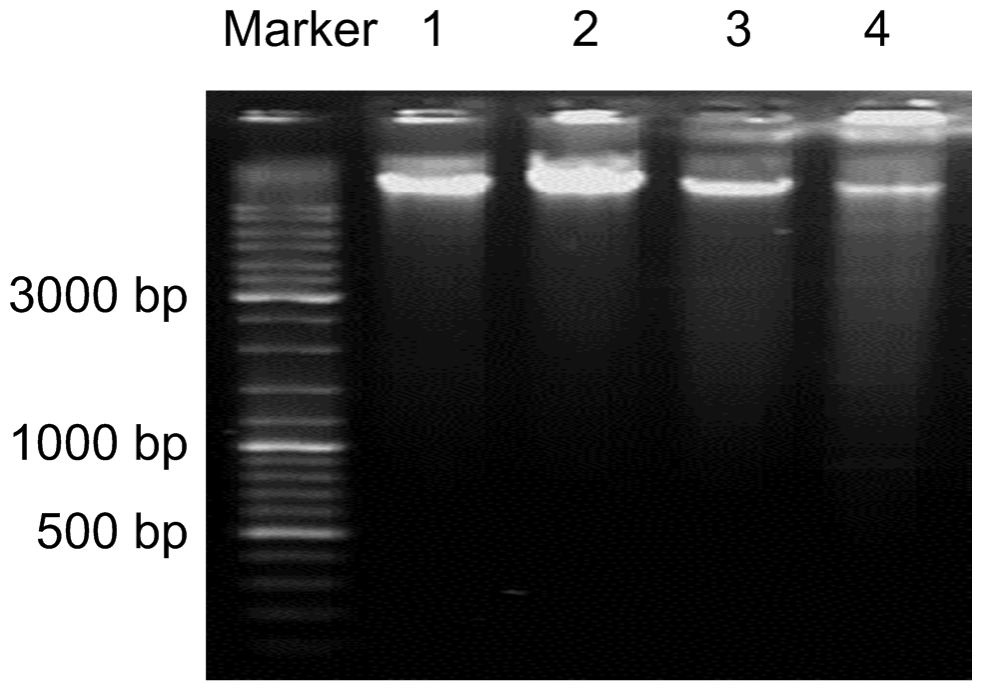
Apigenin induced apoptosis in ANA-1 cells. A typical DNA ladder in ANA-1 cells was observed after being treated with 50 μM apigenin for 48 h. Lanes: 1) control; 2) 12.5 μM apigenin; 3) 25 μM apigenin; 4) 50 μM apigenin.

**Figure 4:**
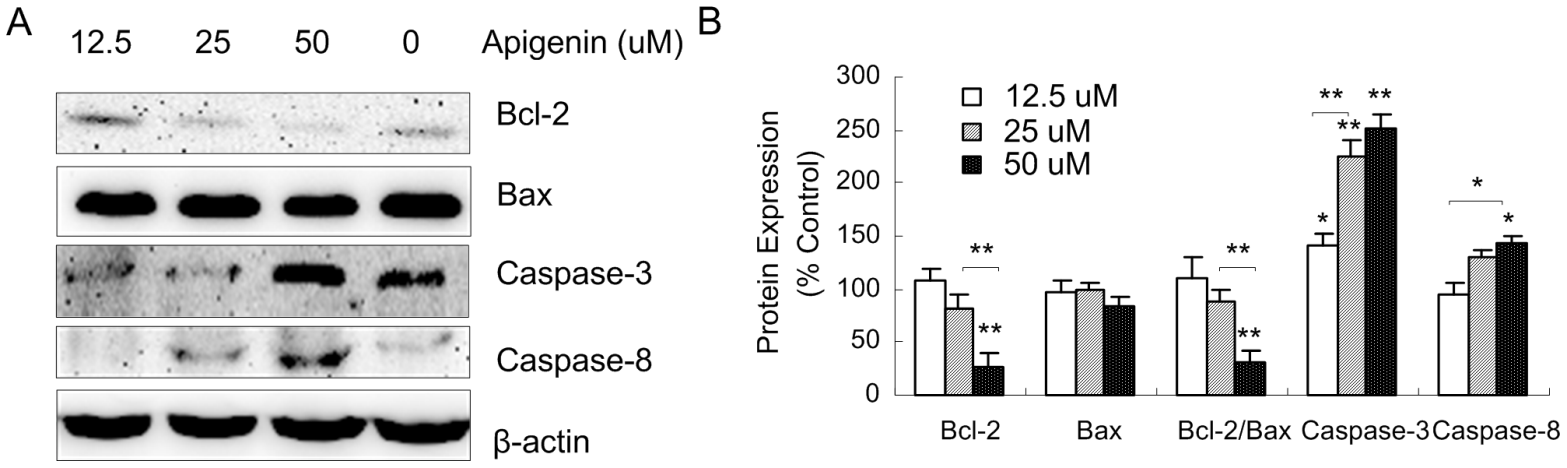
The effects of apigenin on apoptotic and anti-apoptotic proteins in ANA-1 cells. A. Representative western blot results showed Bcl-2, Bax, caspase-3 and caspase-8 expression in the ANA-1 cells after incubation with apigenin for 48 h. B. The relative expression of proteins compared with the control, *, *p*<0.05, **, *p*<0.01, versus the control.

The inhibition of cell viability could result from the induction of apoptosis and/or cell growth arrest. Therefore, we detected the effect of apigenin on apoptosis induction of ANA-1 cells by measuring the accumulation of cells at sub-G_1_, early- and late-stage apoptotic cells; the levels of caspase-3, caspase-8, Bcl-2 and Bax; and the ratio of Bcl-2/Bax. First, we found that apigenin induced ANA-1 cell apoptosis in a dose-dependent manner; specifically, 50 μM apigenin induced the apoptosis of ANA-1 cells by 15% after a 24 h incubation (*p*<0.05) (Fig. 2A–B). Second, we explored the early and late stages of apoptosis in ANA-1 cells after treatment with the indicated concentrations of apigenin for 24 h or 48 h. As shown in Fig. 2C–D, the same concentration gradient of apigenin induced ANA-1 cell apoptosis in a dose- and time-dependent manner (*p*<0.05); both early- (7% and 13%) and late-stage (26% and 49%) apoptosis were induced by 50 μM apigenin at 24 h and 48 h, respectively. The TUNEL assay showed that apigenin could significantly increased DNA fragmentation in the ANA-1 cells (data is not shown) (Fig. 2E). Forty-eight hours after ANA-1 cells were treated with 50 μM apigenin, the typical DNA ladder was observed, while the control had no such ladder (Fig. 3),

Finally, we detected the levels of caspase-3, caspase-8, Bax and Bcl-2 by western blot analysis after an incubation with apigenin for 48 h. As shown in Fig. 4, apigenin treatment (50 μM) increased the levels of caspase-3 and caspase-8. The cells that were treated with 50 μM apigenin for 48 h had significantly increased levels of caspase-3 and caspase-8 (150% and 38%, respectively) (Fig. 4B). The results also showed that apigenin downregulated the expression of anti-apoptotic molecules Bcl-2, but had no significant effect on Bax.

It has been reported that the ratio of *Bcl-2* to *Bax* is another reliable marker for apoptosis [19]. Therefore, we analyzed the ratio of Bcl-2 to Bax using the image analysis software ImageJ (USA). The results showed that apigenin resulted in a significant dose-dependent decrease of Bcl-2/Bax ratio (Fig. 4B).

Taken together, these results demonstrate that apigenin partially inhibited the viability of ANA-1 cells through the induction of apoptosis by regulating the expression of caspase-3, caspase-8 and Bcl-2.

### 3. ROS production induced by apigenin

**Figure 5:**
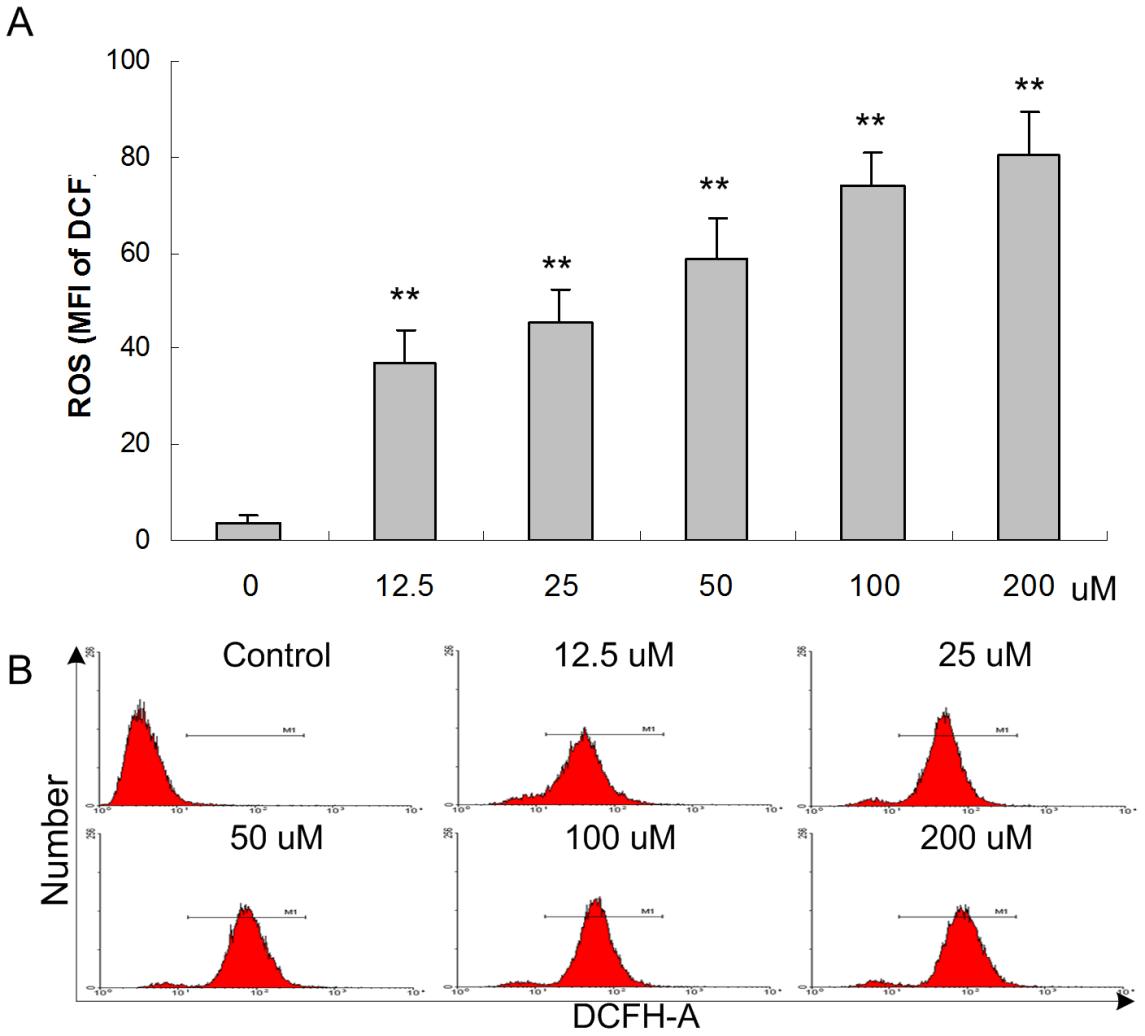
Effect of apigenin on intracellular ROS levels in the ANA-1 cells. The ANA-1 cells were treated with apigenin for 24 h. The ROS levels in the ANA-1 cells. A. The ROS levels are given as the MFI of DCF of cells. The values are given as the mean ± SD. *, *p*<0.05, **, *p*<0.01, versus the control. B. Representative FACS results showed apigenin increased the intercellular ROS levels in the ANA-1 cells after 24 h.

Several researches suggest that ROS generated may play an essential role in the apoptosis induced by apigenin in cancer cells [4]. To clarify whether apigenin effect on intracellular ROS production, ANA-1 cells were subjected to apigenin exposure for different concentration and the ROS level was measured by using the ROS indicator DCF as a probe. As shown in Fig. 5, the levels of ROS (DCF) were increased in the ANA-1 cells treated with 12.5 μM at 24 h significantly (*p*<0.05).

### 4.Apigenin regulates the MAPK signaling pathway in ANA-1 cells

**Figure 6:**
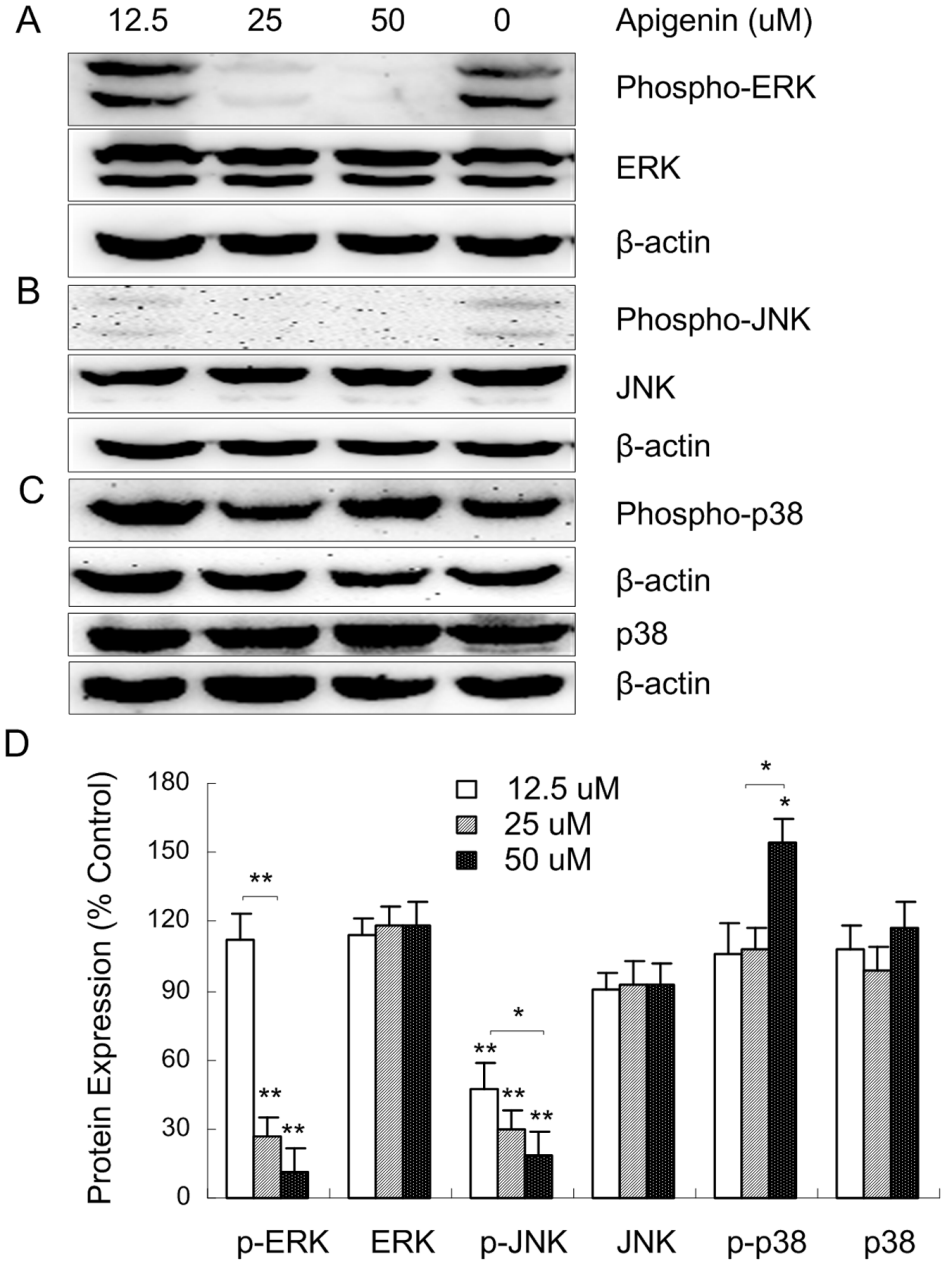
The effects of apigenin on the MAPK pathway in ANA-1 cells. A–C. Representative western blot results showed phospho-ERK1/2/ERK1/2, phospho-JNK/JNK and phospho-p38/p38 expression in the ANA-1 cells after incubation with apigenin for 48 h. D. The relative expression of proteins compared with the control, *, *p*<0.05, **, *p*<0.01, versus the control.

The members of the MAP kinase family mediate a wide variety of cellular behaviors in response to extracellular stimuli. Three of the four main subgroups, the ERK, JNK and p38 groups of MAPK, serve as a nexus for signal transduction and play a vital role in cellular apoptosis [20]. We further examined whether apigenin induced the apoptosis of ANA-1 cells through the MAPK pathway to regulate the expression of apoptosis-related proteins. As shown in Fig. 6 A–C, apigenin (50 μM) treatment decreased the levels of phospho-ERK and phospho-JNK and increased the level of phospho-p38. The cells that were treated with 50 μM apigenin for 48 h significantly reduced the expression of phospho-ERK and phospho-JNK by 89% and 83%, respectively, and significantly increased the expression of phospho-p38 by 54% compared with the control. In parallel, apigenin has no significant effect on the expression of ERK, JNK and p38 proteins (Fig. 6D). The results suggested that apigenin induces the apoptosis of ANA-1 cells may via the MAPK signaling pathway.

## Discussion

The present study demonstrated that apigenin significantly inhibited the cell viability and induced apoptosis in mouse macrophage ANA-1 cells. Furthermore, the results showed that the levels of intracellular ROS, apoptosis-related protein, MAPK expression also changed in ANA-1 cells treated by apigenin. The results suggested that apigenin inducing ANA-1 cells apoptosis most likely by increasing intracellular ROS and regulating the MAPK pathway to inhibit the activity of Bcl-2.

ROS is one of the intracellular second messengers, which affect numerous cellular processes, including metabolism, differentiation and cell proliferation and death by regulating critical signaling pathways [21]. It has been recognized that ROS cause complex and irreversible damage to cellular constituents that impair cellular homoeostasis and elevated levels of ROS can influence central cellular processes, such as proliferation and apoptosis [22], [23]. There is a delicate balance between the production of ROS and the antioxidant defense [24]. Recent researches showed that mild oxidative stress increases proliferation, moderate oxidative stress alters cell physiology to increase the level of protective systems that render the cell more resistant to subsequent insults. Severe oxidative stress results in severe oxidative damage and cell injury, cell senescence and/or cell death [24].

In this report, the results showed that intracellular ROS were elevated significantly compared with the control group in dose-dependent manner in the ANA-1 cells treated by apigenin. Meanwhile, 50 μM apigenin significantly inhibited cell viability and induce DNA damage and cell apoptosis. These phenomena show similar results as previous others' researches and also indicated that ROS may play a key role in apoptosis induced by apignein in the ANA-1 cells.

Previous studies have shown that the activity of Bcl-2 can be inhibited by increasing the activity of caspase 3 and caspase 7, which results in apoptosis in mouse macrophages RAW264.7 [25]. In this study, the results showed that 50 μM apigenin significantly increased the levels of caspase-3 and -8 by 150% and 38%, respectively, in ANA-1 cells (Fig. 4B). Meanwhile, apigenin downregulated the expression of Bcl-2 and decreased the ratio of Bcl-2/Bax (Fig. 4). These results suggested that apigenin induced apoptosis of ANA-1 cells through Bcl-2 pathways.

The MAPK signaling pathways induce either cell proliferation or cell death depending on the cell type and stimulus [26]. Several studies show that the ERK1/2 pathway possesses anti-apoptotic functions, primarily through the increased activation of Bcl-2 and Bcl-XL, which is dependent on the cell type and stimuli [27]. JNKs are indispensable for apoptosis and appear to ensure pro-apoptotic signaling by phosphorylating Bam and Bmf, activate to Bax initiate apoptosis and phosphorylating Bcl-2 in vitro and in intact cells [28], [29], [30]. The signaling of p38 MAPK causes the rapid inactivation of the ERK1/2 pathway and negatively regulates JNK activity [31]. Phosphorylated p38 can also activate a wide range of substrates that are attributed to these phosphorylation events [32].

The studies have shown that apigenin induces apoptosis in a variety of types of cells and exerts a broad range of molecular signaling effects, such as PI3K-Akt in human prostate cancer cells, ERK in RAW264.7 and isolated hepatocytes, JNK in acute carrageenan-induced paw edema and peritonitis [33], [34], [35], [36], [37], [38], [39]. In this study, the results showed that apigenin downregulated phospho-ERK and phospho-JNK and Bcl-2, unregulated phospho-p38 and decreased the ratio of Bcl-2/Bax in ANA-1 cells. The data suggested that apigenin may via the increase of intracellular ROS, the activation of the p38 pathway and the inactivation of the ERK/JNK pathways; and then, inhibiting downstream mediators Bcl-2 expression to induce the ANA-1 cell apoptosis.

In summary, the present study demonstrated that apigenin was able to induce ANA-1 cells apoptosis may through increasing intercellular ROS and regulating MAPK signal pathways. This indicated the possibility of further developing apigenin as a cell immunoregulator in diseases therapy.
